# Conflicts of Interest for Dietary Guidelines Advisory Committee Members: Neither a New Nor Unexplored Issue

**DOI:** 10.1016/j.advnut.2023.07.003

**Published:** 2023-09-13

**Authors:** Mélissa Mialon, Paulo Serodio, Eric Crosbie, Nina Teicholz, Ashka Naik, Angela Carriedo

**Affiliations:** 1 Trinity Business School, Trinity College Dublin, Dublin, Ireland; 2 University of Essex, Colchester, United Kingdom; 3 University of Nevada, Reno, NV; 4 Nutrition Coalition, Washington, DC; 5 Corporate Accountability, Boston, MA; 6 World Public Health and Nutrition Association, Peacehaven, United Kingdom

Dear Editor

We read with great interest the perspective article by Kraak published in *Journal Advances in Nutrition* in 2023 [1] in response to our research article published in *Public Health Nutrition* in 2022 [2]. This perspective paper [1] questions our methods and intentions when writing our manuscript [2]. We gathered information and reported the conflicts of interest (COIs) that all 2020 Dietary Guidelines Advisory Committee (DGAC) members had declared publicly. COI declarations are based on the United States and international best practices.

Kraak [1] claimed that we “did not describe specific research questions or objectives in the methods.” But, in our manuscript, we described the objectives as follows: “In the present study, we therefore aimed to document the COI of DGAC members (in their capacity as scientific experts), and in particular their relationships with industry actors, since these relationships could directly have affected the committee process and decisions.”

Kraak [1] also made unfounded claims about an assumed lack of information regarding the methods used by us. Contrary to Kraak’s [1] assertion, we did report on the industry actors covered in our study, stating, “corporate actors from the food, drink and pharmaceutical industries, as well as third parties working with them such as trade associations or front groups. We included pharmaceutical companies, because some sell infant nutrition products and often offer devices or drugs that compete with food-based solutions to chronic diseases.” Regarding what Kraak [1] unspecifically described as “funding thresholds,” our manuscript covers all information declared by DGAC members, with no restriction on time. Moreover, Kraak [1] questioned our methods for data analysis. As part of standard journal practices, we noted that “All the COI compiled in our database were independently verified by PS, MM and AC.” We also included “supplemental evidence tables for independent verification” when we submitted our manuscript to *Public Health Nutrition* as a source for any reader to obtain further details on our data and analysis. The final version of our manuscript with the supplementary material is yet to be published *Public Health Nutrition*, for reasons beyond our control, despite our request to do so.

Kraak [1], in addition, questioned the fact that “no detailed information” about the CoI reported were included in our manuscript. We did not have that information as it was not disclosed by DGAC members in their own reporting of COIs. In addition, contrary to the claims made by Kraak [1], we made no further interpretations about the reported COIs of DGAC members, as emphasized in the Discussion section of our manuscript. We noted, “based on the information we collected, we could not say whether a COI had led to bias, as this was beyond the scope of this article.”

Kraak [1] then claimed that if we had examined “the institutional context in which the DGAC members were engaged,” it “would have revealed that the NASEM standards for transparency and scientific integrity were met.” This is inaccurate. The only National Academies of Sciences, Engineering, and Medicine (NASEM) recommendation relevant to our manuscript is that the United States Departments of Agriculture (USDA) and Health and Human Services (HSS) should be “publicly posting a policy and form to explicitly disclose financial and nonfinancial biases and conflicts of committee members” [3]. Our investigation did not find that these disclosures had been publicly posted, as noted in our manuscript. Another team of researchers also searched for the same disclosures but without success [4]. Whether the USDA-HHS met other NASEM standards for “transparency and scientific integrity” was outside the scope of our manuscript. Kraak [1] indeed suggested that we should have included “the institutional COI policies and procedures used by the USDA and HHS to select the committee.” However, the rules and procedures of the USDA and HHS are irrelevant to our manuscript. We rather sought to report on ties between DGAC members and commercial actors, as we stated in our manuscript, “Nor did we study the process for developing the DGAC scientific report and how COI and other forms of influence by industry actors may have impacted its writing; this could be the subject of future analyses.”

Our publication and its dissemination on social media were characterized, without substantiation, as “digital vigilantism” by Kraak [1], which is a defamatory statement.

Based on these unfounded claims, Kraak asked *Public Health Nutrition* for a correction of our manuscript and publication of an apology to DGAC members as Kraak [1] claimed, “Publishing information that harms an individual’s reputation without evidence is called defamation.” It is unclear to us how the information that is already made public by DGAC members themselves could bring any harm to their reputations.

Interestingly, Kraak [1] misinterpreted and cherry-picked information from our funding statement, where we declared “The sponsor had no input in the study design; in the collection, analysis, and interpretation of data; and in the decision to submit the article for publication.” Contrary to what Kraak [1] claimed, we noted right after that sentence that, “N.T., Director of the Nutrition Coalition, had input in the report's writing.” We were not funded by any other source for the study. So, we stand by our declaration, which is aligned with *Public Health Nutrition*’s policy, developed by the International Committee of Medical Journal Editors, where COIs to be declared include “all relationships/activities/interests (…) that are related to the content of your manuscript” [5].

Discussion regarding COIs of DGAC members is neither a new nor an unexplored issue. Framing it as a radical, targeted, or defamatory topic seems uninformed and loses sight of the context, which is why this issue had already received attention from the research community and the media [[6], [7], [8]] and was even the reason why a United States Senator recently called for suspending work of the 2025 DGAC [9]. Documenting potential corporate influence on public institutions and processes is essential for a healthy democracy. These institutions are currently undergoing a crisis of trust. Enhanced transparency can only ultimately help strengthen the faith of the public in the government.

## Funding

The authors report no conflicts of interest. Melissa Mialon reports financial support was provided by Health Research Board, Ireland [grant ARPP-2020-002]. N.T. is the Director of the Nutrition Coalition, U.S., a non-profit group that has a policy not to accept any industry funding.
